# Hospital in-reach family-centred social prescribing pilot for children with neurodisability: mixed methods evaluation with social return on investment analysis

**DOI:** 10.1186/s12913-025-12329-0

**Published:** 2025-01-30

**Authors:** Laura Gordon, Megan Hastry, Angela Bate, Katie Gordon, Emily Greaves, Simoni Dimitriadou, Tim Rapley, Anna Purna Basu

**Affiliations:** 1 Population Health Sciences Institute, Newcastle University, Level 3, Sir James Spence Institute, Royal Victoria Infirmary, Newcastle upon Tyne, NE1 4LP UK; 2 School of Psychology, Newcastle University, Newcastle upon Tyne, UK; 3 Nursing, Midwifery and Health, Northumbria University, Newcastle upon Tyne, UK; 4 Biomedical Sciences Suite, Newcastle University, Newcastle upon Tyne, UK; 5 Social Work, Education and Community Wellbeing, Northumbria University, Newcastle upon Tyne, UK; 6 Paediatric Neurology, Great North Children’s Hospital, Newcastle upon Tyne, UK

**Keywords:** Social prescribing, Neurodisability, Child, Hospital setting, Community setting, Link worker

## Abstract

**Background:**

Social prescribing link workers support individuals to engage with community resources, co-creating achievable goals. Most schemes are community-based, targetting adults. Vulnerable populations including hospitalized children with neurodisability and their families, could also benefit from social prescribing.

**Aims:**

To pilot a hospital-initiated social prescribing service for children with neurodisability and their families; to explore its feasibility, acceptability and undertake social return on investment (SROI) analysis.

**Methods:**

Mixed-methods cohort study with SROI analysis. We recruited children aged < 16y with neurodisability, identified during inpatient stays, their parents/carers and siblings. Participants received link worker support for 6 months, extending beyond hospital discharge. Pre- and post-intervention pilot data covered profile of needs (Support Star), quality of life (EQ5D/CHU-9D), wellbeing (WEMWBS/CORS) and financial strain. We undertook 22 qualitative observations of family/link worker interactions and 39 in-depth interviews with families, link workers and healthcare professionals. Together these data were analysed within a SROI to establish the costs and social value generated.

**Results:**

Of 48 families supported by the service, 25 were recruited to the evaluation (26 children, aged 10 m-15y; 4 siblings; 36 parents). Baseline quality of life and wellbeing indices averaged below population norms. Link workers were highly effective at supporting families (only 6/151 goals unmet). Unmet need decreased by 6 months (Support Star, *p* < 0.001).

Families reported having felt overwhelmed when trying to adjust to new ways of life post diagnosis/discharge before link worker intervention, with little support to navigate non-medical needs. Parents, link workers and health care professionals found link worker support invaluable for making community services accessible. Families then felt more connected to their communities, and less isolated, with increased belief in their self-efficacy. Families and healthcare professionals felt that the duration of support, and eligibility criteria, should be extended. Inputs to deliver the service for 1 year (49 families) were estimated at £74,736: outcomes for the 18 families studied were estimated at a value of £205,861.

**Conclusion:**

Hospital in-reach social prescribing is feasible, acceptable, and addresses a range of otherwise unmet needs of children with neurodisability and their families, showing a positive SROI. Other vulnerable patient groups could also benefit from this approach.

**Trial registration:**

ISRCTN23306751 (2.8.22).

**Supplementary Information:**

The online version contains supplementary material available at 10.1186/s12913-025-12329-0.

## Background

Social prescribing involves “link workers” helping those with health and social needs to engage with supportive community resources, addressing social determinants of health [1]. Social prescribing is attracting interest globally [2] and is endorsed by the National Health Service (NHS England) across the age spectrum within primary care settings as part of personalised care. Addressing unmet non-medical needs is postulated to improve clinical outcomes by reducing competing demands, thus freeing up time, money and energy, reducing stress and improving wellbeing. This could lead to reduced emergency appointments, improved adherence to medications and routine appointments, resulting in reduced healthcare costs [3].

Social prescribing interventions in adults have shown benefits to wellbeing and quality of life [4]. Such schemes have led to cost savings in secondary care. Analysis from a large cohort of adults with multiple long-term conditions in the Ways to Wellness programme in Newcastle showed 27% lower secondary care costs compared with a matched group [5]. Evidence of reduced use of primary care services is summarised in a recent report for the National Academy of Social Prescribing [6].

Social prescribing for children is under-investigated [7], underdeveloped [8], and underfunded [9], despite evidence of benefit [10]. For example, the Charity Barnardos showed that their scheme for children and young people in Cumbria improved mental wellbeing and was cost effective [9]. Most studies have focused on young people (children aged 14 and over) and young adults rather than younger children [11], though a few community-based studies are exploring social prescribing to support the mental health of children [12]. An NHS toolkit, accessed through the StreetGames website, has been developed for those intending to deliver social prescribing for children and young people. Given the known association between poverty, family adversity and adverse child outcomes [13], social prescribing initiatives for children and families have real potential for positive impact.

Social prescribing approaches are likely to be of benefit to children with neurodisability and their families [14]. Neurodisability can be defined as ‘a group of congenital or acquired long-term conditions that are attributed to impairment of the brain and/or neuromuscular system and create functional limitations. A specific diagnosis may not be identified. Conditions may vary over time, occur alone or in combination, and include a broad range of severity and complexity. The impact may include difficulties with movement, cognition, hearing and vision, communication, emotion, and behaviour’ [15].

Children with neurodevelopmental impairments e.g., cerebral palsy, epilepsy and autism, form the largest group of children with disability in the United Kingdom (UK). Over half of families with a child with complex healthcare needs, such as those arising from neurodevelopmental impairments, have financial difficulties; nearly half express unmet non-medical service needs, and a third have difficulty accessing non-medical services [16]. Parental financial constraints impact participation of their children [17]. Access to medical services is also affected: non-attendance at outpatient appointments is more common in children with cerebral palsy whose families are socio-economically disadvantaged [18]. Parents of children with disabilities want support in finding services to support the individual needs of their child, siblings and the whole family [19]. Social prescribing could help address this need. Finally, the health and psychosocial functioning of parents of children with neurodevelopmental disorders is adversely affected [20]; this further indicates the need for a family-centred approach to support.

Initiating non-medical support for hospitalized children with neurodisability and their families during their hospital admission could help reduce length of stay, reduce readmission rates, improve outcomes for patients and families, and allow specialist staff to redirect support to link workers, improving work efficiency. This approach has not been adequately explored in hospital settings [21]. USA inpatient screening identified that children with medical complexity had an increased likelihood of a positive screen for social risk factors [22]. Children’s hospitals provide a valuable setting to identify and address health inequalities, opportunistically and/or systematically [22, 23]. Our prior consultation work with stakeholders including young people, parents of children with disability [24], healthcare providers and managers indicated a high level of interest in a hospital in-reach social prescribing service for children with neurodisability.

## Aim

To pilot a hospital-initiated social prescribing service for children with neurodisability and their families.

To explore the feasibility and acceptability of the service and conduct a social return on investment (SROI) analysis of the associated costs and outcomes.

## Methods

### Design and setting

The study used a mixed methods design with SROI analysis to evaluate an innovative family-based social prescribing pilot intervention in a children’s hospital in North-East England between August 2022 and February 2024. During this time, two part-time link workers (just under one full time equivalent), employed by an external organisation (Ways to Wellness, an innovation hub tackling health inequalities in the North East and North Cumbria), provided support to children with neurodisability and their families identified within the hospital. The pilot was called SPACE CYP (Social Prescribing And Community rEsources for Children and Young People), or the “SPACE Pilot”. During the setup phase, Ways to Wellness advertised the posts, shortlisted, interviewed and recruited two link workers specifically for the project, in consultation with the chief investigator (CI) and hospital management. Essential qualifications for the post were: a relevant “level 3” (post 16 years) qualification in mental health/wellbeing or youth work or equivalent relevant experience and good overall topic-based training covering a range of health and wellbeing disciplines. Excellent communication skills and knowledge of the local community as well as experience of working in a social prescribing or similar role were among other essential attributes.

Link workers obtained honorary contracts with the hospital Trust. Training was provided in social prescribing, youth work, and the full mandatory NHS training program including data protection and safeguarding. Training in Good Clinical Practice for Clinical Research was also provided, as was training in the use of assessment tools including the Support Star (Triangle); other relevant training opportunities were taken up as they arose. Mentoring and supervision were provided from both Ways to Wellness and the CI with frequent team meetings to discuss progress. Link workers compiled a comprehensive database of local services relevant to the client group as well as setting up referral pathways. A secure data environment was set up to store data regarding client contacts.

In line with the Medical Research Council's complex intervention guidance [25], this service was an adaptation of an existing intervention to a new context (hospital in-reach, family-based service for a specific patient group). It included assessment of feasibility and acceptability of the intervention and evaluation design. A logic model was created and informed the pilot evaluation (Figure S1).

### Participants, inclusion and exclusion criteria

We recruited children with neurodisability, their carers, and siblings. Eligible children were aged under 16 years, had complex chronic needs related to neurodisability, and were hospital inpatients at the Great North Children’s Hospital, Newcastle upon Tyne (admitted under any team) at the time of identification. The NHS defines a child with complex needs as one who “has been diagnosed with an illness, disability or sensory impairment and needs a lot of additional support on a daily basis” [26]. A chronic complex condition in children is one that “can reasonably be expected to last at least 12 months unless death intervenes and to involve either different organ systems or one organ system severe enough to require specialty pediatric care and probably some period of hospitalization in a tertiary care centre” [27]. There were no specific eligibility criteria regarding length of hospital stay. Eligible children and families were resident in the North of Tyne/Gateshead region as it was felt that link workers would reasonably be able to maintain oversight of relevant facilities in this region. Children were excluded if they were too medically unwell for a social prescribing intervention to be appropriate at the time (with the offer of support at a more appropriate or convenient later date); and if they did not meet the eligibility criteria. We considered whether participants were already involved in a research study to make sure that they would not be, or feel, overburdened by research commitments; and to ensure that taking part in more than one study would not interfere with any study findings. We also recruited link workers and healthcare professionals working in the children’s hospital for their views and feedback on the service, aiming for a wide range of professions and varying levels of experience.

Ethical approval was provided by NorthWest—Greater Manchester Central Research Ethics Committee (ref 22/NW/0110). Children and families not meeting criteria for entry to the study (and/or not providing informed consent) were still supported by the service. For example, where families did not live in the North of Tyne/Gateshead region, link workers would meet with them, discuss their needs, and signpost to local resources. The trial was registered on 2.8.22 as ISRCTN23306751.

### Sample size

We aimed to recruit 30 children and families to the study, depending on caseload complexity. The sample size was chosen pragmatically, based on anticipated capacity of the service and numbers likely to meet inclusion criteria and participate.

### Participant identification and recruitment

Potential participants were identified by ward staff who were part of the existing care team. Ward staff had access to the eligibility criteria for the service and were able to contact link workers with any queries. Potential participants were provided with information about the service research study, initially through a flyer. If interested, they were given an information sheet about the study and had the opportunity to have questions answered. Children could provide assent where appropriate in addition to obtaining parental consent. As support was provided to the family unit in addition to the primary participant, consent was sought for parents and carers for their own participation, as well as parental consent or written assent for any siblings of the primary participant as appropriate. Written informed consent was obtained prior to any study procedures taking place.

### Intervention

During the intervention phase, link workers met with the child and family to establish individual profiles of need, working collaboratively with them to identify goals and ways to address these. Link workers connected families with relevant community services where available, and provided them with personalised and facilitative support over a six-month period. During this time, they tailored support to the needs of the family, and reviewed progress, informally at 3 months and more formally at 6 months. There was no control group: all those recruited were offered link worker support for ethical reasons. We created a TIDIER [28] checklist summarising the key features of the intervention (Table 1).

**Table 1:** Intervention summarised using TIDIER checklist

Name	GNCH pilot
Why	Children with neurodisability and their families have high levels of unmet need. Social prescribing is a plausible solution to addressing this
What materials	Support star (Triangle) used by link workers to gather information about domains and levels of need, at baseline and follow up. This forms the basis of a conversation about unmet needs in a range of domains Directory of community resources and services, curated by link workers
What procedures	Unmet needs mapped; participants referred to services/community resources to address these needs and supported to attend where needed
Who provided	Link workers – with relevant life experience, personal qualities and values to undertake the work. Appropriate training undertaken on social prescribing and supplemented with courses to fill in gaps in experience as well as hospital mandatory training. Shadowing with hospital team. Supervision and line management. Employed by external organisation with honorary contracts with the Hospital Trust
How provided	Individual, face to face session at baseline. Ongoing follow up by face to face/telephone/virtual means, with face-to-face reviews at 3 and 6 months
Where	Children identified during inpatient hospital stay Baseline assessments in hospital or in the community post discharge e.g., patient’s home Engagement with link workers (up to 6 m) and community services within the North of Tyne and Gateshead Integrated Care Partnership (indefinite) continues after discharge from hospital
When and how much	Intervention tailored to need within the 6-month period
Tailoring	Personalised intervention, adapted to address unmet need
Modifications	If recruitment through inpatients slow, extend to outpatients
How well (adherence)	Documentation of link worker contact, goals set, goals met and how: in-depth interviews with families at the end of the intervention

### Data collection/assessments

Data were collected to assess feasibility outcomes with the intention that at least 30 children and families took up the offer of the scheme and that at least 2/3 of families engaged with and reported benefits from the service. We collected qualitative data for feedback regarding acceptability of the scheme to families, referrers and link workers. We also piloted assessments of wellbeing and quality of life as detailed below.

#### Process data to understand pathways and throughput.

Baseline data collection including demographics and contact details for families were collected as part of standard care for link worker interventions. We collected data on the number of referrals, uptake, numbers declining, deemed inappropriate/ineligible or discontinuing engagement; demographics; goals (including whether they were met and if so, how), and the nature of the work undertaken by link workers.

#### Qualitative data to understand features of an optimal service

In-depth interviews were undertaken with families (parents and children including recruited siblings where possible) to explore their experiences of the service. Interviews were offered to all participants at 3 and 6 months and were undertaken face to face or remotely according to participant preference. A topic guide was developed to guide but not restrict the flow of discussion (Supplemental data S2). Broadly, the first interview aimed to understand the context of the child and family; their experiences of meeting the link workers and of the support offered; and any suggested changes to the service. The second interview continued with these themes but also explored what had changed due to the intervention as well as thoughts around closure/discharge from the service. Interviews were supplemented by qualitative observations of interactions between the link workers and families, and of situations in which children and families were engaging with community services to which they had been referred, as well as interviews with link workers and healthcare professionals working in the children’s hospital for their feedback and views on the service.

#### Quantitative pre- and post-intervention data

Pre and post intervention data were collected to pilot assessments covering the following domains: profile of needs; quality of life and wellbeing, and specific data relevant to the population under study including financial strain.

##### Profile of needs

The Support Star (*Triangle Consulting Social Enterprise Limited*) was used by the link workers as a tool to start conversations with the family about how they were managing. The Support Star assesses seven domains of functioning (physical health, study and work, “doing what matters to you”, money, friends and relationships, home and family, and emotional wellbeing). Scores of 1–5 respectively represent “not being able to respond”, “taking it in”, “trying to respond”, “finding a way through” and “managing well”. The Support Star was designed for use with young people facing serious illness and was chosen as it is responsive to change even for service users whose health condition may be deteriorating [29]. The young person’s version of the Star was used. In practice, link workers used the Star as a tool to capture how the family was managing overall with respect to each domain. However, if there were two affected children with neurodisability in one family who were in the study, a separate Support Star was used for each child, so that the focus on needs and goals in relation to each child was clear. The rationale for each score in each domain was documented. The score helped link workers to understand the priorities of the family and to support them in setting personalised goals around areas of unmet need. After 6 months of support, link workers categorized whether these goals were fully met, partially met, unmet or no longer relevant.

##### Quality of life

Generic quality of life (QoL) measures were piloted to estimate both child and parental changes in QoL outcomes. The Child Health Utility Instrument (CHU9D) [30, 31] is a generic preference-based measure of health-related quality of life (HRQoL) designed for children and young people aged 7–17 years though a proxy version can be used for younger children. There are 9 items, each with 5 response levels (1 being the best outcome for each level) based on a recall period of one day. Individual scores were weighted using the UK Adult Tariff [31] to give a HRQoL score between 0.33 and 1. Normative scores for Australian adolescents (*n* = 500) [32] show a mean HRQoL of 0.930 (std 0.083) for males and 0.932 (std 0.085) for females.

The EuroQol-5 Dimension (EQ5D-5L) [33] was used to capture parental HRQoL. The EQ5D-5L has five items, each with 5 response levels, where 1 is the best outcome response for each level. Scores were weighted using a tariff to give a number between 0 and 1. The visual analogue scale (VAS) score was also obtained to indicate parental self-reported health on the day of assessment on a scale from 0–100. Normative English general population data by age were published in 2023 [34].

##### Wellbeing

The Childhood Outcomes Rating Scale (ORS) [35] was piloted as a tool to assess wellbeing in children. This is a linear visual analogue scale used with four items (“me, family, school, everything”), with a simplified child- friendly version initially designed for those age 6–12 years (CORS) but valid up to age 15 years [36]. For children aged 5 years and under the YCORS can be used to allow a self-report choice amongst 4 facial expressions from sad to happy. There is a maximum total score of 40 (best outcome). Scores below 28 are of concern. Community-based normative data in children aged 10–15 years showed a mean score of 31.4 (std 7.69) [36].

The Warwick-Edinburgh Mental Wellbeing Scale (WEMWBS) was used to assess parental mental wellbeing [37]. This has 14 items each with 5 response levels where 5 is the best outcome response for each level. The mean score in the English population was 51 with standard deviation of 7 [37].

Three questions were put to parents as a gauge of self-perceived financial strain, taken from the US National Longitudinal Surveys question bank [38]. These related to how often the household put off buying something they need because they don’t have enough money (5-point scale); difficulty paying bills in the last 12 months (5-point scale); and financial situation at the end of each month (e.g., “just enough to make ends meet”: 4-point scale).

### Data analysis

Descriptive statistics were used to summarise quantitative data. Interviews and observations were recorded verbatim, transcribed and anonymised for analysis. Observation and informal debriefs involved the production of anonymised field notes. All qualitative analyses were conducted according to standard rigorous procedures. We used procedures from first-generation grounded theory (coding, constant comparison, memoing), from analytic induction (deviant case analysis) and constructionist grounded theory (mapping). We undertook independent coding and cross-checking, and a proportion of data was analysed collectively in data clinics with the core research team where people shared and exchanged interpretations of key emerging issues [39].

#### Reflexivity

The CI acknowledged that her involvement in identifying the need for and setting up the service could lead to bias. This, and her role as a clinician could create a perceived power imbalance and affect what interview participants felt comfortable sharing. Therefore, in-depth interviews were undertaken and first analysed by non-clinical members of the research team, who had not been involved in the design or setup of the service. Interviews with families and link workers were undertaken by LG, a research assistant with a degree in psychology and prior interviewing experience. Interviews with healthcare professionals were undertaken by SD as part of a Psychology Masters degree. Training and supervision were provided. Interviewers were aware that participants could find it distressing to reflect on the challenges they had faced, and knew how to signpost to support. Likewise, the CI offered support and space for reflection to the interviewers. To enhance data exploration, both LG and MH (psychology undergraduate student who undertook training qualitative methodology prior to commencing the work) coded transcripts, compared codes, and agreed on themes, with supervision from the CI and TR (professor with expertise in qualitative research methodology). Sense checking of findings was also undertaken through discussion with link workers during team meetings and in follow up interviews with parents.

### SROI Analysis

SROI analysis followed six key stages: establishing scope and identifying key stakeholders, mapping outcomes, evidencing outcomes and assigning value, establishing impact, calculating SROI and reporting, using and embedding [40].

#### Establishing scope and identifying key stakeholders

Stakeholders included the SPACE CYP participants (children with neurodisability), their parents & siblings with unmet non-medical needs and NHS England, as identified using inclusion and exclusion criteria detailed earlier. Link workers delivering the intervention and healthcare professionals involved in care of the families were also included.

#### Mapping outcomes

Data from qualitative interviews with stakeholders were used to refine and shape the theory of change outlined in Figure S1, which demonstrates the relationship between inputs, activities, outputs, outcomes and impacts.

#### Evidencing outcomes and assigning value

Data on outcomes were obtained via questionnaires from participants using the measures outlined above including Support Star, CHU-9D, EQ5D-5L, CORS, WEMWBS, and financial strain. Data were collected at baseline and follow-up at 6 months. Detailed outcome data were obtained from the 19 children (18 families) recruited to the study over a 14-month period between 9th August 2022 and 10th October 2023 and who completed follow up. Baseline and follow-up questionnaires were compared to identify changes in levels of unmet non-medical needs and number of goals met, children’s quality of life and mental wellbeing, parent’s quality of life and mental wellbeing and financial strain.

Outcomes that resulted in direct financial gains or losses (such as annualized increases in benefits) could be valued directly. Social outcomes without market values were assigned values indirectly using the Housing Associations’ Charitable Trust (HACT) Social Value Calculator version 4 [41] which uses established wellbeing valuation methods to assign monetary values to social outcomes [42]. Inputs were valued in terms of the total cost of running the SPACE CYP intervention for one year (for the full service which supported 49 families). This included: staffing costs, equipment costs, project marketing, licensing and software.

#### Establishing impact (Attribution, Deadweight Displacement)

To avoid over-claiming the benefits, we accounted for attribution, deadweight and displacement. Deadweight is the proportion of outcomes that primary stakeholders would have experienced, regardless of engaging with the SPACE CYP project. Displacement refers to the proportion of activities that participants had to give up to engage with the SPACE CYP project, which may have contributed towards their wellbeing. Attribution acknowledges the proportion of outcomes that could be attributable to factors other than the SPACE CYP project. Displacement and attribution were calculated retrospectively, using data from qualitative interviews. To calculate attribution, qualitative interviews with families were screened to establish whether families were supported by other professionals/services prior to link worker support. To calculate displacement, interviews were screened and key terms such as ‘Give Up’, ‘Stop’ and ‘discontinue’ were utilised. To avoid over-claiming standard deadweight, reductions were taken from the HACT Mental Health and Social Value Calculator. To calculate deadweight for financial outcomes, ranges were taken from five social prescribing studies which included SROI analysis [43–47]. Quality assessments were undertaken on these studies following a quality framework tool [48].

## Results

### SPACE CYP Pilot service

Sixty-three children from 62 families were referred into the service over a 14-month period between 9th August 2022 and 10th October 2023. Three families were excluded because the child referred did not have a neurodisability; two families could not be supported because of language barriers and challenges accessing a translator. Thirteen families lived outside of the pilot service area and were signposted to local support. The remaining 48 families were offered support for 6 months in the first instance. This represented 49 children with neurodisability (mean age 7y3m, range 5 m to 15y; 33 male), 9 siblings, and 65 parents (123 clients in total). 45 families spoke English as their first language and three had a different first language (French, Arabic and Javanese).

For 22 families the referrer was a nurse and for 21 cases a consultant. The other 5 referrals were from a physiotherapist (*n* = 3), an occupational therapist (*n* = 1), and staff from the Bridges hospital in-reach school (*n* = 1).

### Pilot study patient flow

Twenty five of the referred families (26 children with neurodisability, mean age 6y10m, range 5 m to 15y, 17 male, 36 parents and 4 siblings (mean age 6y3m, range 21 m to 9y, two of whom were older than their sibling with neurodisability) were recruited to the pilot evaluation. The proportion of children with neurodisability from ethnic minority groups (5/26) was representative at both a local (North-East England) and National level when compared to 2021 ONS Census data. 5/25 families had postcodes in the most deprived Index of Multiple Deprivation national decile.

Three families withdrew from the study prior to the baseline data collection. One was due to death of the child, one family felt the study was too much to take on at the time, and one was lost to follow-up before baseline data was collected. At baseline, data was collected from 22 families, including 23 children with neurodisabilities and 26 parents.

Each family received at least six months of link worker support between baseline and end-line. In this time, four further families withdrew from the study. One was due to death of the child, and three were lost to follow-up prior to final data collection. Final data was collected from 18 families (19 children with neurodisabilities and 20 parents): this met our feasibility criterion regarding evidence of engagement of at least 2/3 of families recruited.

**Fig. 1:**
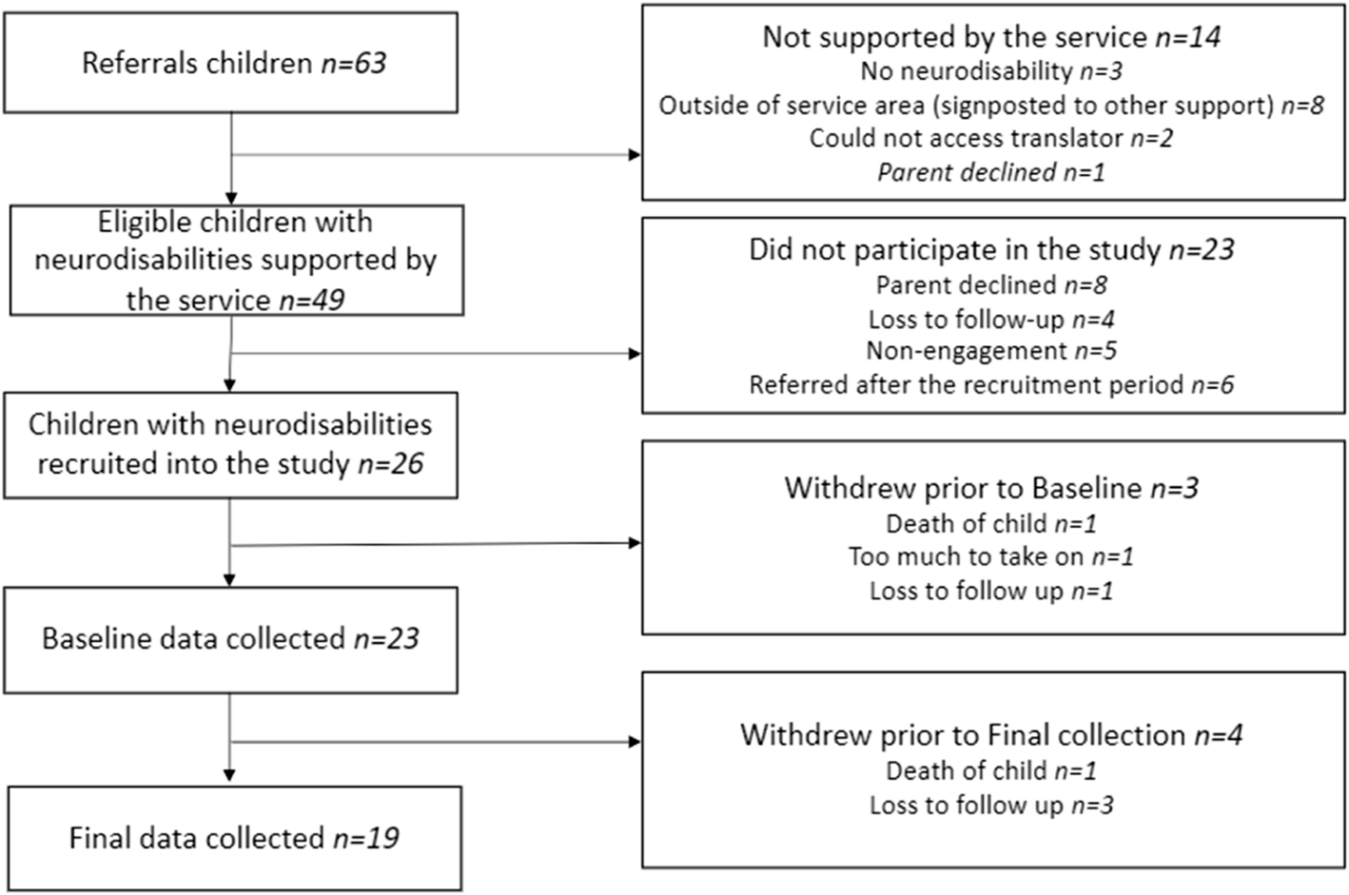
Flow of children with neurodisabilities through the service and pilot evaluation. Note that parents and siblings were also supported: details are in the manuscript text

Figure 1 shows the flow of children with neurodisability through the service and pilot evaluation. Table 2 shows the participants by family structure. In 9 of 20 two-parent families, only one parent consented to participate. There were 5 (20%) single parent families in the pilot study.

**Table 2:** Child and family participants recruited to research study (pseudonyms)

Child (gender; age at start)	Recruited Parents	Recruited Sibling	3 m Interview	6 m Interview
Ashley (F, 4y)	Chloe, Barry		Yes (Chloe)	Yes (Chloe)
Amina (F, 1y)*	Blessing, Umar		No	No
Iris (F, 10 m)*	Karen, John		Yes (Karen & John)	Yes (Karen & John)
Lewis (M, 6 m)*	Mandy, Nigel		Yes (Mandy)	Yes (Mandy)
Olivia (F, 11y)	Paula		No	No
Ryan (M, 15y)	Sarah		Yes (Sarah)	Yes (Sarah)
Toby (M, 8y)	Andrea		No	No
Vince (M, 6 m)*	Wendy, Xavier		Yes (Wendy)	Yes (Wendy)
Jackson (M, 13y)	Angela		Yes (Angela)	No
Bobby (M, 10 m) *	Christina		No	No
Doug (M, 3y 10 m)	Ellie		No	No
Fergus (M, 1y)*	Georgia, Harry		No	No
Ahmad (M, 8y)	Umi	Lina (F, 6y)	Yes (Umi)	Yes (Umi)
Luke (M, 6y)	Melissa, Nick	Mason (M, 9y)	Yes (Melissa & Nick)	Yes (Melissa & Nick)
Parker (M, 1y)*	Robert		No	No
Sadie (F, 7y)	Tanya		No	No
Vaughan (M, 6y) *	Whitney		Yes (Whitney)	Yes (Whitney)
Yasmin (F, 14y)*	Zara		No	No
Fiona (F, 1y) *	Harriet, Mark	Leo (M, 1y)	Yes (Harriet)	Yes (Harriet)
Charlie (M, 4y)	Rachel	Josh (M, 7y)	Yes (Rachel)	Yes (Rachel)
Billy (M, 13y)	Esme, Reggie		Yes (Esme)	No
Ollie (M, 9y)	Alice		No	No
Amara (F, 2y)	Ola, Ibrahim		Yes (Ibrahim)	Yes (Ibrahim)
Molly (F, 13y) Alfie (M, 13y)	Rose, Myles		Yes (Rose & Myles)	No
Samuel (M, 14y)	Phoebe		Yes (Phoebe)	Yes (Phoebe)

Asterisks indicate families with children with new neurological presentations or children age < 2y (emerging neurological presentations)

### Staff participants

Table 3 summarises the 11 staff participants recruited to the study. Participants from the following backgrounds were interviewed: paediatric consultants (general/diabetes; neurology/neurodisability; rheumatology), children’s physiotherapy and occupational therapy, children’s nursing (1 community and 2 hospital-based), a hospital in-reach link worker, a teacher and a paediatric neurology trainee.

**Table 3:** Staff recruited to the study for interviews

Participant Pseudonym	Gender	Occupation	Time in role
Audrey	Female	Children’s physiotherapist	8 years
Brad	Male	Consultant in general paediatrics and paediatric diabetes	9 years
Daphne	Female	Consultant in paediatric neurodisability	7 years
Eliza	Female	Children’s community nurse	18 years
Frida	Female	Children’s nurse & coordinates discharge	20 years & 7 months for coordinating discharge
Gloria	Female	Consultant in paediatric rheumatology	10 years
Kaitlin	Female	Occupational therapist specialising in cerebral palsy	25 years
Lionel	Male	Senior registrar in paediatric neurology	10 years
Melissa	Female	Teacher on children’s wards	Not stated
Miley	Female	Nurse in charge of paediatric neurology ward	29 years
Gemma	Female	Hospital based Link worker	9 years of link work experience

### Baseline assessments

Index cases in the study were significantly impacted by their neurological conditions. The exact nature of the neurological conditions varied, but included cerebral palsy, epilepsy, autism, developmental delay, genetic syndromes, spina bifida, neuromuscular and neurodegenerative disorders. 22/26 index children had feeding difficulties. Only 3/26 could walk independently, though 8/26 were aged < 2y. Young age and/or significant communication difficulties necessitated proxy reporting for almost all assessments undertaken.

At baseline, CHUD data were completed for 22 children. Mean weighted proxy score was 0.725 (s.d. 0.129), with population normative mean 0.89. Mean weighted EQ5D-5L score for parents at baseline (*n* = 27/36) was 0.82 (s.d. 0.14), lower than population norms (mean 0.905); mean VAS score was 68.7 (s.d. 17.9).

CORS could only be completed in 16 children at baseline. At baseline, 7 (43.8%) of CORS scores were below the cutoff for concern (28), and the mean CORS score was 27.9 (s.d. 5.46). Mean WEMWBS score (26 parents) was 42.7 (s.d. 9.58), versus a UK population norm of 51.6. At baseline, 7 parents had a WEMWBS score of 41–44 (possible or mild depression range) and 9 parents scored < 41 (probable clinical depression).

Regarding financial strain, only 3 families reported never having to put off buying essential items due to lack of money over the previous 12 months. Only 5 families reported never having difficulty paying bills. 5 families reported having some money left over at the end of the month (only one of these scored as “more than enough money left over”).

Median Support Star scores at baseline in relation to 23 children (22 families) were 3/5 for physical health, work and school, “doing what matters to you”, money, and emotional wellbeing, and 3.5/5 for friends/relationships and home/family.

Thus, the assessments piloted were all workable except that the item relating to school in the CORS was problematic given the young age of most participants. The YCORS for younger children does not map neatly to the CORS, so could not be used as an alternative.

### Goals identified at baseline and goals met

Link workers identified 151 goals amongst 23 families in the pilot evaluation. Table 4 shows the number of goals sorted by domain and primary beneficiary (child/parent/sibling/whole family). Table 5 gives examples of goals, presented in the same format. Of the 151 goals set at baseline 110 were completed, 24 partially completed, and 11 were no longer relevant. Only six goals were not met – three were for additional financial support which was not available (for specialist equipment, childcare costs and general support); three were around parental needs (physical, emotional) but the parent was unable to engage with these due to the child’s medical needs.

**Table 4:** Numbers of baseline goals by domain and primary intended beneficiary

Domain	Child (proxy)	Parent	Sibling	Whole family	Total
Physical health	5	2	0	1	8
“Doing what matters to you”	22	5	3	14	44
Money	2	1	0	17	20
Home/family	7	4	0	14	25
Friends/relationships	0	12	1	0	13
Emotional wellbeing	0	24	4	2	30
Study and work	1	8	1	1	11
**Total**	**37**	**56**	**9**	**49**	**151**

**Table 5:** Examples of baseline goals by domain and primary intended beneficiary

Domain	Child (proxy)	Parent	Sibling	Whole family
Physical health	Conductive Education Wheelchair sports Sensory support	Exercise Advocacy in raising concerns re factors affecting child’s physical health		Accessible play parks
Study and Work		Advocacy re absence from uni Employment advice (ACAS)/ Business advice/ Carers rights at work/Back to work courses	Advocacy re regarding mitigating circumstances for sibling	Nursery funding application
“Doing what matters to you”	Accessible activities (outdoor activities/holiday clubs/swimming/ horse-riding/ cycling/ wheelchair activities	Driving/motability courses Parent wellbeing activity; Recovery College; “Disability expo”	Sibling activities e.g. play group ADHD youth club	Blue badge/Radar key/ Buggy & Wheelchair transport Holiday activities/ Accessible days out
Money	Eyegaze technology funding Activities/clubs where personal assistant goes free	Childcare costs		Food bank, MAX card, DLA/Carers Allowance/ Direct payments advice/ Finance review Advocacy e.g., support to apply for funding; energy bill dispute
Emotional wellbeing	Safe sensory chew toy		Pre-bereavement support; Young carers	Pre-bereavement support; Emotional support during PICU
Friends/ relationships		Online/Face to face peer support including specific groups e.g. ADHD Making friends	Sibling peer support	Peer support for total parenteral nutrition at home Meeting other families with children with cerebral palsy
Home/family	New bed/suitability of environment for child with disability Obtaining disability social worker	Visa advice Respite care		Accessible family holiday; Housing/garden repairs/items. Advocacy in obtaining house assessment report

### End of study assessments

**Fig. 2:**
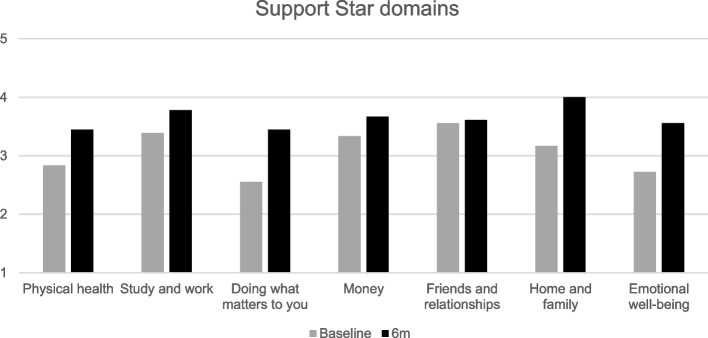
Support Star mean scores (*n* = 19 children) at baseline versus endline, by domain

Support Star data were available in relation to 19 children from 18 families at the final time point; these scores were compared with the same children at baseline. There was a mean increase in total scores of 3.94 points (median increase 4 points), with the domains “doing what matters to you”, “home and family” and “emotional wellbeing” showing the greatest increases and “friends and relationships” showing the least change. Figure 2 gives an overview of the changes by domain. Wilcoxon signed rank analysis (2-tailed) demonstrated a significant change in total scores from baseline to 6 months (z = 3.34, *p* < 0.001). Support Star scores improved for 15 of 19 children, showed no change for 3 children and decreased for one child. Review of these four cases provided an explanation: all four of the children had a significant deterioration in physical health over this time and one subsequently died.

In contrast, quality of life and parental wellbeing measures EQ5D-L5 (including domains), CHU9-D (including domains), and WEMWBS did not change significantly over the 6-month period (though 6/19 parents showed a meaningful improvement in scores of at least 3 points on the scale). The mean reported level of financial strain also did not change significantly over the 6-month period (Table 6). CORS did show statistically significant improvement but was only applicable to 13 children; in three of these the change was 5 points or more (reliable) and in two it was also considered clinically significant (i.e., it also crossed the clinical cutoff). Thus, the Support Star was the most sensitive indicator of change in outcomes in the pilot study, capturing changes in outcomes in domains related to goals set.

**Table 6:** Baseline versus endline data for quantitative assessments

Domain	Assessment	Number with baseline and endline data	Mean at baseline (s.d.)	Mean at endline (s.d.)	Mean difference (s.d.)	Test statistic and p (2 tailed)
**Unmet need**	Support Star (high score is better)	19	21.8 (4.11)	25.8 (4.39)	3.95 (3.73)	z = 3.34 *p* < 0.001*
**Quality of Life,** **Child**	CHU9D total (low score is better)	18	21.9 (5.59)	19.9 (7.93)	-2.00 (9.05)	z = -0.996 *p* = 0.319
	CHU9D utility (high score is better)	18	0.717 (0.126)	0.767 (0.147)	0.05 (0.159)	t = 1.34 *p* = 0.199
**Quality of Life, Parent**	EQ-5D-5L index (high score is better)	19	0.800 (0.159)	0.817 (0.121)	0.0163 (0.128)	t = 0.540 *p* = 0.597
	EQ-5D-5L VAS (high score is better)	19	68.7 (19.0)	69.0 (16.5)	0.263 (22.1)	t = 0.052 *p* = 0.959
**Feedback on progress, Child**	CORS (high score is better)	13	28.1 (5.39)	31.5 (4.57)	3.33 (4.16)	z = 2.90 *p* = 0.004*
**Wellbeing, Parent**	WEMWBS (high score is better)	19	42.5 (9.55)	44.3 (9.39)	2.00 (9.79)	t = 0.891 *p* = 0.385
**Financial strain**	Financial strain (low score is better)	18	7.83 (2.07)	7.50 (1.98)	-0.33 (1.64)	z =—0.644 *p* = 0.519

Results for participants with data at baseline and endline

### Qualitative analysis

We undertook interviews with 15 families. Of the 10 families who did not take part in interviews, two had children who died; two commented that their child’s poor physical health precluded their involvement at that time; one was lost to follow up and another had to negotiate a complex social situation. Three of the remaining families set and achieved or partially achieved all their goals and one family set two goals but did not achieve them. Those who were interviewed found link worker support very helpful: however, they did describe barriers to engagement, detailed below, which in their cases were overcome.

- A. Context preceding link worker support: Illness, overwhelm and constant hypervigilance

Families reported a range of medical issues faced by their children, and the impact on the physical, mental, social and financial wellbeing of the family. The number of appointments with healthcare professionals was overwhelming, as were the frequent, lengthy and unpredictable hospital admissions. Inpatient stays were often in the context of new significant neurological events, either as an initial presentation or an exacerbation or deterioration of a known condition.

… he’s gone from being really active, running around, to not being able to hold his weight or walk or stay upright. He deteriorated with his mobility pretty quickly after being diagnosed. So, it was really scary to watch.

(Whitney, IV-F9, 32)

In addition to the stress and anxiety caused by illness in their child leading to “constant hypervigilance”, prolonged hospital inpatient stays caused significant disruption to family life and reduced access to routine community engagement and support.

You have a whole different community being a hospital mam… … being stuck in the hospital I missed that whole part of having a baby, coming home …, and you meet other mums at baby groups, and you learn about things going on.

(Harriett, IV-F13, 15)

The transition from hospital to home after such an admission was described as overwhelming. Parents who had faced this transition on their own in the past reflected that in-reach link worker support would have been valuable. Healthcare professionals concurred, and felt that link workers could facilitate hospital discharge, helping families to “find ways of adjusting and going forwards at home” (Kaitlin, IV-H07, 87). Some parents reflected on memories of the early days and months after their child had first been discharged some years previously. In addition to fears about medical management and everyday care of their child, they described having felt lost when trying to adjust to new ways of life. They felt there had been no clear path to finding the support they needed. They had to tackle changing housing needs, juggle caregiving commitments with work and/or apply for welfare support, and source specialist equipment and practical tools. The complexity of their child’s medical needs created significant barriers when trying to access support, including difficulties when leaving the house, limited childcare opportunities and finding services to accommodate their unique needs. Parent carers who left their previous job due to their child’s health reported that their mental loads were significantly increased by their caring responsibilities.

Families also reported having experienced gaps in service provision. Some (e.g., those with young children) did not meet eligibility criteria for a disability social worker; others were reluctant to accept support from statutory services, perceiving the potential for stigma. Early Help support required re-referral whenever new issues emerged. Overall, services were not always felt to be accessible or to address their needs.

Parents prioritised their children’s needs but neglected their own wellbeing. For example, some children had complex physical care needs (e.g. gastrostomy feeding) which parents became skilled at, but which were time consuming and which they could not easily delegate. Attendance at frequent medical appointments was also time consuming. Prioritising these needs had financial impacts e.g. where one parent gave up their job to become a full-time carer. Parents described having limited time for self-care such as physical activity despite recognizing the benefit to their own wellbeing. Social isolation, feelings of overwhelm and exhaustion were also described.

Parents worried about the impact on siblings.

…And I think there was a little bit of guilt for feeling frustrated by the fact that [sibling] does miss things when Luke’s ill and there is more responsibility on him than a normal 10-year-old.

(Melissa, IV-F24, 253)

The impact on siblings included emotional wellbeing challenges due to their circumstances and the requirement of parents to focus their time and energy on looking after their child with neurodisability.

- B. Overcoming barriers to engagement

Many families reported having no prior knowledge of link worker services and didn’t initially understand the role, despite receiving information leaflets and explanations from referrers.

… it was really quite vague in the hospital …, a lot of the nurses and stuff were saying it was a social worker, and then it was like, “it’s not a social worker, it’s duh-duh-duh,” and I was like, “that’s a bit confusing.”… I think it might have been Gemma [link worker] when she came then discussed that “we want to see if the support you have already, if there’s any gaps that need filling.”.,

(Mandy, IV-F1, 212)

Other barriers included scepticism borne out of a history of prior negative experiences with other services, and a feeling that their situation was too complicated for improvements to be possible. Some parent carers reported having been in “survival mode” for so long that they had no energy to contemplate longer term goals and felt overwhelmed at the thought of engagement with another service. Others felt they should be coping alone and that seeking help was an admission of failure. Trust and belief were built through experiencing positive changes facilitated by engagement with the link workers.

I was worried at first. When the nurses came into hospital and said there was this project and you wanted to come and see us, I thought, “Oh, God.” (Laughter) I have never been a one for joining groups and things … So, I thought, “Oh, no. What is this going to be?” And then, once I started chatting to you, I realised that “Actually, this is something that is missing.” (Angela, IV-F4, 65).

- C. Situating link workers within the hospital: Family-centred holistic care

Link worker in-reach into the hospital created opportunities for face-to-face meetings with families. This helped build familiarity and trust, and reduced the social isolation felt because of prolonged periods spent in isolated cubicles with otherwise predominantly medically focused contacts. The co-location of link workers at the hospital also allowed for greater collaborative work with healthcare professionals that families liaised most closely with, and allowed prompt and timely initiation of support.

Healthcare professionals reported encountering a range of unmet non-medical needs of children and young people and their families including issues related to isolation, inclusivity and accessibility, self-management of their condition, mental health, socioeconomic factors and lack of awareness of existing services. One healthcare professional (‘Gloria’), referring to children with rheumatological disorders, commented that “like any other group of people looking after long-term conditions, we’ve got another group who really, really struggle, and many of whom may be neurodiverse”.

Healthcare professionals encountered challenges in signposting families to services due to their own lack of awareness of appropriate services, lack of time and biases inherent in their role. The link worker role was perceived as identifying and addressing a range of psychosocial factors, for example:

… it would take the pressure off. Particularly nurses, therapists in our service, because they are often the first port of call for families. Families will tell them things that make them feel very sad and they don’t know where to send that family. Sometimes an awful lot of a Band 7 nurse, physio’s time is taken up trying to find where and how to help this family … I just think we are not doing any of this very effectively. If you had a person whose job it was to understand these services outside of the hospital, they would do it more effectively. It would take less time, it would cost less money, and it would work better for families. (Gloria, IV-H06, 234).

Link worker support was seen by healthcare professionals as contributing to more family centred care, resulting in reduced caregiver burden, improved health outcomes for children and young people and reduced pressures on healthcare systems. Financial and operational challenges in setting up a new service were acknowledged.

- D. Ensuring support tailored to the clients’ needs

The nature of support varied as needed. Some families benefitted from ‘enhanced signposting’, noting that link workers provided tailored information not collated on websites. Some received help with completing referral forms for access to services. Some parents needed a link worker to go with them to attend a community group for the first time.

A lot of the time, I will go, I will support people to attend for the first time because I think it’s a huge positive in getting over the first hurdle. I think, with a lot of people, signposting is not enough. You have to do, what I call, signposting plus. So, like signposting plus the added extra of the support … So, we will support people to attend places, to build confidence and break down barriers.

(Gemma, link worker, IV-L06, 116).

Some services had specific eligibility criteria which inadvertently excluded certain groups from applying despite having capacity to expand. Link workers directly brokered expansion of eligibility criteria of several services for client benefit. They did so by leveraging an in-depth knowledge of families and community services to problem-solve and advocate for solutions from their place of relative impartiality.

- E. Attributes of a successful link worker

Link workers were viewed by parents as trusted, empathetic, impartial, non-judgmental, and intuitive:

Because there are times when these things happen where you just don’t know where to turn. And it is good to have somebody who is there who is able to have the time and resources to be able to try and think where you need to turn, to give you that little bit of information and advice. Because it’s so lacking, it really is… I think it’s a very, very valuable service.

(Sarah, IV-F12, 392–394).

Link workers were felt to have very good knowledge and understanding of the types of challenges parents might face, and of available and relevant community support. They were flexible, approachable, proactive and resolute in matching services to need. Due to the level of specialization required for this patient population, mainstream and inclusive services were often insufficient for families’ needs but link workers secured opportunities that families had never thought possible.

- F. Actualising change: outcomes after link worker support

In addition to achievement of goals (Tables 4 and 5), parents felt listened to, supported and motivated to tackle challenges that had previously defeated them, such as reapplying for support which had initially been refused; or daring to attend a new group with their child.

I spoke to Helen [link worker] yesterday and I told her about the play group and she’s like, “do you want me to give you a pep talk on Thursday morning?” I’m like, “yes please, because I will cancel.” She’s like, “no, you’re going. I can tell you really want to go, so you’re going. I’m gonna ring you and give you that pep,” I’m like, “okay.”

(Wendy, IV-F5, 127).

They valued the delight in their children when they could access new community activities and were keen to share information about sources of support with others facing similar difficulties, e.g., through peer groups. Over time they felt more connected to their communities, and less socially isolated, with increased belief in their self-efficacy to sort problems out in the future and in some cases to support others in similar situations.

… at least I’ve got those pathways now, haven’t I? And I know if maybe that group doesn’t exist anymore, I might be able to find another one that’s similar to it because I know who’s on it and I’ve got an email address, you know what I mean? That sort of thing.

(Sarah, IV-F12, 408)

- G. Moving on

Some families felt ready for discharge at 6 months; some felt relieved that they could still access help if new problems arose (“link worker in reserve”; some needed ongoing help. In practice, the “link worker in reserve” model was often used. A link worker commented on the on the types of situation leading to discharge versus ongoing support.

“It just depends what we’ve done for people. If it’s been quite a straightforward thing where people have got a lot of support in place and they only wanted bits and bobs of signposting and linking in with a couple of things, it’s easier to end because you can just say to people, “We’ve completed the goals that we set. You’re welcome to re-approach us in the future….”

… where it’s a family that has been much more complex and they’ve been through quite a lot of trauma and you’ve supported with a lot of different goals, those sorts of endings are much more tricky because, I guess, you want to feel like you’ve put enough in place for them to be able to navigate those systems without their link worker, which is the whole point.”

(Gemma, IV-L06, 222; 224).

- H. Suggestions for the service: “support more groups of children, for longer”

Parents and professionals knew of other families in similar situations who would benefit from similar support and felt that the eligibility criteria should be broadened. It was felt that overall this type of support was missing for children with long term conditions and their families, and would be invaluable. The evaluation challenge of capturing meaningful improvements in the context of fluctuating health issues of the child, was recognised.

I think the last time we saw [name of link worker], we were struggling a lot. Things were really, really bad. And she’d asked us to redo the circle of where you are…And I was quite upset that that makes it look bad like she isn’t doing what she’s supposed to be doing. But it wasn’t that. It was just life.

(Melissa, IV-F24, 459–460).

It was acknowledged that 6 months was a relatively short period of time for provision of link worker support given the enormity of challenges faced by some children and families, so flexibility regarding the duration of input as well as the nature of input was proposed. Whilst the link worker service was viewed very positively, other barriers remained, e.g. the cost and logistics of travel to some services to which families had been referred.

### SROI analysis

Costs for delivering the social prescribing intervention over 12 months were estimated to total £74,736 or £1525 per family supported by the service. These are presented in Table 8. Direct financial outcomes are presented in Table 9. Many of the goals set by link worker and participants resulted in direct financial outputs. Some of these were beneficial to families and resulted in financial gains whilst a few led to financial losses.

**Table 7:** Total annual costs per family for SPACE CYP

Cost Category	Annual Cost £	Annual Cost per Family supported (*n* = 49) (£)
**Staffing** (2* part time link workers)	51,558	1052.20
**Training**	3000	61.22
**Travel**	9791	199.82
**Equipment** Computers • total • annual^a^ Wellbeing Star License MIS information (fixed) • total • annual^b^ MIS license (variable) • monthly • annual	(9437) 1887 1560 (11,400) 1140 (150) 1800	- 38.52 31.84 - 23.26 - 36.72
**Marketing**	4000	81.63
**Total Cost**	**74,736**	**1525.21**

^a^assuming computing equipment will depreciate after 5 years

^b^MIS license is for 10 years

**Table 8:** Direct financial outcomes per family (by child)

Study ID	Goal	Financial Gain (£)	Financial Loss (£)	Deadweight (%)	Net Value (£)
**C10001**	Swimming	X	336	25	-252
**C10001**	New Bed	258	X	25	194
**C10001**	DLA	5,644*^a^	X	25	4,233
**C10004**	Baby items	90	X	25	68
**C10004**	Bedroom environment	72.97	X	25	55
**C10009**	Activity centre	X	56	25	-42
**C10009**	Family Holiday	745	X	25	559
**C10009**	Paediatric first aid course	209	X	25	157
**C10009**	Counselling	450*^b^	X	25	338
**C10012**	Counselling	450*^b^	X	25	338
**C10012**	DLA	3,783*^a^	X	25	2,837
**C10012**	Employment advice & carers allowance	4,258*^c^	X	25	3,194
**C10017**	Blue Badge	X	10	25	-8
**C10017**	Radar Key	X	5.49	25	-4
**C10017**	Family holiday	1027	X	25	770
**C10020**	Dehumidifier	139.97	X	25	105
**C10020**	Finance review	6000	X	25	4,500
**C10033**	Advocacy for housing	1500	X	25	1,125
**C10033**	Rain cover & hood	209	X	25	157
**C10033**	Activity centre & transport	55.40	X	25	42
**C10050**	Activity centre	20	36	25	15 -27
**C10050**	Carers allowance	4,258*^c^	X	25	3,194
**C10065**	New Bed	392.50	X	25	294
				**Total Value**	**£21,842**

^a^Middle and higher rates taken from UK Government website [49]

^b^Counselling costs taken from Leo’s Neonatal website [50]

^c^Carers allowance rate taken from UK Government website [51]

Wellbeing outcomes were valued using the HACT Mental Health Social Value Calculator. Wellbeing data collected through the WEMWBS was mapped on the SWEMWEBS and the Mental Health Social Value Bank was used to value changes in participant scores. A standard reduction of 27% was applied to account for deadweight as recommended by HACT [52]. Total social value was calculated for each adult participant (Table 7). All other outcomes were valued using the HACT Social Value Bank.

**Table 9:** Mental health social value calculator

Study ID	Baseline Score	Follow-up Score	Value Change £	Value – 27% Deadweight £
P10003	25	29	1,255	916
P10011	28	26	-652	-476
P10013	15	18	2,616	1,910
P10018	14	31	25,856	18,875
P10025	20	21	3,488	2,546
P10021	21	22	0	0
P10031	14	15	9,369	6,839
P10034	26	27	652	476
P10037	20	18	-5,306	-3,873
P10041	25	26	0	0
P10045	19	24	5,383	3,930
P10051	26	26	0	0
P10057	24	27	1,933	1,411
P10061	26	28	652	476
P10063	25	23	-1,281	-935
P10067	30	26	-1,255	-916
P10069	20	22	3,488	2,546
P10070	22	21	0	0
P10064	18	18	0	0
			**Total Social Value**	**£33,725**
			**Total Social Value per Family**	**£1,775**

We measured 13 outcomes from the Social Value Bank, evidenced using the goals data (from Support Star) and data from the qualitative interviews. For example, one parent set a goal to take up regular volunteering: this goal was completed and evidenced in their interview transcript, resulting in an outcome of ‘Regular Volunteering’. ‘Financial comfort’ was evidenced via the financial strain questionnaire and mapped directly on to the HACT outcomes and values. For all outcomes, participant scores were averaged, and boundary scores calculated, to assess whether the participant’s change was significant enough to be included. Deadweight was taken directly from the HACT Social Value Bank. Attribution and displacement were estimated from qualitative data (as presented in the methods above). We estimated attribution at 18%, suggesting that 18% of any change could be due to other support besides link worker support delivered through this intervention. Displacement was estimated at 0%, because SPACE CYP did not appear to displace any other activities. Table 10 illustrates the number of people experiencing changes for each outcome, and the resulting social value, when deadweight and attribution were considered.

**Table 10:** Social value generated by stakeholder group

Stake-holder	Outcome	Financial Proxy £	Number experiencing the outcome	Deadweight %	Attribution %	Net social value £
SPACE CYP Participants	Able to obtain advice locally	2,773	18	9	18	37, 245
	Private outdoor space	3,052	2	0	18	5,005
	Afford to keep house well-decorated	13,984	2	22	18	17,888
	Financial comfort	17,118	6	31	18	58,112
	Goes to youth clubs	720	10	12	18	5,195
	Member of a social group	1,734	10	1	18	14,076
	Frequent moderate exercise	3, 662	8	12	18	21,139
	Secure job	10,569	1	34	18	5,719
	Frequently walk or cycle	5,447	1	5	18	4,243
	Vocational Training	3,648	1	3	18	2,901
	Satisfactory landlord maintenance	2,606	1	38	18	1,324
	Rectification of mould	6,495	1	0	18	5,325
	Regular volunteering	5,344	1	30	18	3,067
NHSE	Reduced number of bed days ^a^	600	14*	25	18	6,300
					**Total Social Value**	**150, 294**

^*^Refers to the reduction in number of bed days rather than number of people experiencing the outcome

^a^Jones, K. & Burns, A. (2021) Unit Costs of Health and Social Care 2021, Personal Social Services Research Unit, University of Kent, Canterbury. https://doi.org/10.22024/UniKent/01.02.92342

The estimated total value, combining values from the direct financial, wellbeing, and social outcomes, attributable to the SPACE CYP project in one year is presented in Table 11, at £205,861.

**Table 11:** Total social value

Social outcomes	Mental Health outcomes	Direct Financial outcomes
£150, 294	£33,725	£21,842
		Total Value = £205, 861

The total value of outcomes for the 19 children (18 families) in the evaluation study (£205,861) divided by the value of inputs required to deliver the SPACE CYP project for one year including support for all 49 families (£74,736) generates a SROI ratio of £2.75 of social value generated for every £1 spent.

## Discussion

Social risks are common in the paediatric inpatient setting [22] but are not systematically addressed in routine practice. Children with disabilities face significant inequities and have recently been recognised by the National Institutes of Health as a health disparity population [53]. Our study demonstrates feasibility and acceptability of a hospital in-reach social prescribing service for children with neurodisability and their families.

The number of children referred to the service exceeded our estimates; however, not all these met eligibility criteria for the study. This was largely due to their residing outside of the geographic area that we pre-specified. Our rationale for specifying an area of residence was to ensure link workers would have a good knowledge of services within this area, and so that travel to events or services with families, if required, was feasible. The challenge within a pilot study was that there was no similar service in nearby regions, so link workers supported families outside of the study area as part of the service. Retention of children and families through the study met feasibility criteria, with high levels of benefit in terms of achievement of goals set. Qualitative feedback showed strong support for the service from families and healthcare staff, with interest from both groups in extending eligibility criteria to other patient groups and other settings such as outpatients. We intend to explore these options in our ongoing work.

One consideration regarding eligibility is whether all children in hospital should be screened for unmet social needs and support offered on this basis. In the United States, screening for social determinants of health has been mandated by the Centers for Medicare and Medicaid services from 2024, though a range of screening tools are used in practice [54]. Systematically screening for social determinants of child health in “well child” community reviews and referring as appropriate improves use of community resources by families [55]. This approach could help with prioritisation of families for support but would need exploration and evaluation in a UK setting.

Data from qualitative interviews and questionnaires highlighted the importance of a hospital in-reach approach, identifying families who had “fallen through the net”, with high levels of unmet social needs. Families had little time or energy to address these needs on their own, in the context of having a child with complex chronic health needs. The co-location of link worker staff and healthcare professionals facilitated communication and referrals, also helping referrers to understand the link worker role [56]. Qualitative interviews captured a range of views on why needs had not been met by existing services – these included: lack of parental “headspace” to think about what was required and search websites for relevant information, discomfort about making time for their own wellbeing in the face of illness in their child, mental exhaustion associated with the effort of applying for support, and sometimes a feeling of perceived stigma at the involvement of statutory services. There were some initial barriers to link worker input in our pilot, with the need to understand the potential value and gain trust in the link workers, but these were easily overcome.

The value of provision of “in-person service navigation” over an active control such as written information has been demonstrated in previous research involving children in primary care settings [57], showing improved social situation and overall health. Given the level of burnout seen in parents of children with complex chronic needs [58], link worker support is likely to be even more valuable in this population, even if the potential to benefit overall health may be lower and the impact of the underlying medical condition is large.

A family-based approach was appropriate in our setting for several reasons. Firstly, most children in the study required their parents to advocate for them due to young age or communication challenges. Secondly, there was an impact on the financial, emotional and mental wellbeing of parents and siblings, which is recognised in families of children with medical complexity [59]. Thirdly, many goals were at a family level, or were set on the premise that improving parental wellbeing was important in its own right and in supporting parents to be able to fulfil their critical and challenging roles in looking after their children with additional needs [20]. In theory there could have been conflicting interests in relation to goals, but we were not aware of any outcomes from goals which disadvantaged the child.

One important finding was the lack of prior knowledge of the link worker role amongst hospital staff and patients, despite this role being relatively well established in community services in the UK. This may reflect the unique situation of our link workers within secondary and tertiary care children’s services in contrast to most social prescribing schemes which involve link workers supporting adults within the community. However, it is also symptomatic of a wider issue of awareness and buy-in reported elsewhere, including in GP surgeries where the relative priority of making referrals to link workers has sometimes been viewed as low in comparison to addressing clinical situations [60]. More work is needed to increase awareness and buy-in from clients and referrers. The term “social prescribing” is counter-intuitive and often not initially understood [61]. A recent study surveying views on social prescribing found that healthcare professionals felt they would make more referrals if they had a better understanding of the link worker role [62]. Co-location of link workers with referrers can help to resolve this issue by increasing visibility of the service and fostering greater collaborative work between professionals [63, 64]. There is a clear need to continue to promote knowledge and understanding of the link worker role where services are in their infancy. Short videos describing our pilot service from the point of view of different stakeholders were produced as an output of this project [65]. The use of infographics to explain the link worker role could be further explored. Feedback mechanisms to better convey the outcomes of social prescribing referrals to referrers could also help build trust in the service.

Practical arrangements such as training, line management and support/supervision for the link worker in-reach service were generally feasible but critical to consider. Whilst there are national recommendations for link workers working with children and young people (StreetGames toolkit), the hospital setting provides additional challenges in terms of the emotional burden shared by families with children experiencing serious illnesses, sometimes terminal; accessibility of services to children with mobility issues; and working with children with significant communication difficulties. The link workers could be considered to have a “specialist” role, supporting a group of children with complex chronic health needs and their families: equally, it is important that the boundaries of link worker roles are respected and that social work, crisis mental health teams, palliative care and indeed acute hospital services are used where appropriate.

The SROI analysis highlights that social prescribing administered by link workers generates social value for children with neurodisability and their families, with an SROI ratio of at least £2.75 for every £1 spent. This was calculated based on inputs related to supporting 49 families, and outputs (social value) related to the 19 children (18 families) in the detailed evaluation study. It is reasonable to assume that further social value was gained by the other families supported by the service who were not part of the detailed evaluation study. However, it is also likely that the social value per family is not comparable between the two groups. This is because the service supported some families who were not eligible for the detailed evaluation due to living outside the recruitment area, and link workers were more limited in what they could offer under these circumstances.

The findings suggested that social value was generated to participants through increased mental wellbeing, increased material benefits, increased social connectivity and increased knowledge of services, which aligns with the outcomes proposed in the theory of change. Data from qualitative interviews highlighted that participants attributed 82% of the observed benefits towards the SPACE CYP project. The theory of change also hypothesised that there would be cost saving to the NHS and fewer barriers to discharge following link worker support. This was also met where link workers facilitated a faster discharge resulting in cost saving to the NHS (£6,500 in bed days). The SPACE CYP project also resulted in cost savings for families through sourcing holiday funding, referral to charities for counselling and items/activities paid for by Ways to Wellness, an external organisation employing the link workers.

The study design lacked a control group. We considered that leaving a vulnerable population with high levels of social needs without support for the purposes of control data would be unethical. The issue was moderated by accounting for deadweight, attribution and displacement. In the future, it may be appropriate to supply participants with a follow-up questionnaire, rather than using data from qualitative interviews.

The main barrier to ongoing service provision is funding. The two main components of this are the relative lack of link worker provision for children and young people in contrast to adults [9] and the focus on community-based social prescribing services. There is also the issue of who should be addressing health inequalities in children’s hospitals – this is perceived as “everyone’s business” but “no-one’s responsibility” [66], which makes it more challenging to find the most appropriate source of funding. We maintain that a hospital in-reach service is justified, based on our findings, and ultimately links patients back up to community services which would otherwise be bypassed.

Apart from the qualitative interviews, outcomes for families were best captured by the Support Star and the number of goals met, by domain. Others have also struggled to identify appropriate outcome measures for social prescribing and have found goal-based outcomes valuable in practice [67]. Mental wellbeing and quality of life outcomes might require a longer duration of support for statistically significant benefit to be seen. However, deterioration in the child’s physical wellbeing has a large adverse effect on outcomes, as was discussed by one of the parents. This is a major challenge in demonstrating the benefit of link worker support for children with significant health needs and their families. Traditionally, a randomised trial with a control group would be an appropriate method but the sample size would need to be very large to compensate for the wide range of pathologies encompassed by “neurodisability” and the range of interventions as driven by individual needs. Furthermore, a trial would only be appropriate if we were in equipoise regarding the potential for benefit. We are in no doubt that families have benefitted from link worker support: the focus for future research should encompass health service delivery and economic aspects. However, there remains a place for a new, family-based wellbeing outcome measure for use to capture baseline and post-intervention scores.

Strengths of the study include collection of a large amount of detailed information for each family. Limitations include the small sample size and single site, as well as the necessity to use proxy measures for most child outcomes. It was not possible to capture the patient voice in interviews, largely due to communication challenges faced by the children recruited, but we intend to do this in future studies.

In summary, our pilot service and evaluation demonstrate the feasibility and acceptability of a hospital in-reach social prescribing link worker service for children with neurodisability and their families, evidencing a high level of baseline unmet social needs and proof of concept that families can be effectively supported to achieve mutually agreed goals. Next steps include broadening the service scope to support children with other forms of medical complexity; multi-site evaluation to demonstrate scaleabilty and spread; and working with policymakers and commissioners to achieve widespread delivery.

## Supplementary Information

Supplementary Material 1: Figure S1: SROI logic model.

Supplementary Material 2. S2: Topic guides for qualitative interviews.

## Data Availability

The data that support the findings of this study are not openly available due to reasons of sensitivity and are available from the corresponding author upon reasonable request.

## References

[1] Drinkwater C, Wildman J, Moffatt S. Social prescribing. BMJ. 2019;364:l1285. 30923039 10.1136/bmj.l1285

[2] Morse DF, Sandhu S, Mulligan K, Tierney S, Polley M, Chiva Giurca B, et al. Global developments in social prescribing. BMJ Glob Health 2022; 7. 10.1136/bmjgh-2022-008524 PMC9115027 35577392

[3] Gurewich D, Garg A, Kressin NR. Addressing social determinants of health within healthcare delivery systems: a framework to ground and inform health outcomes. J Gen Intern Med. 2020;35:1571–5. 32076989 10.1007/s11606-020-05720-6 PMC7210348

[4] Polley M, Chatterjee H, Asthana S, Cartwright L, Husk K, Burns L, et al. Measuring outcomes for individuals receiving support through social prescribing. London: National Academy for Social Prescribing; 2022.

[5] Case T, Drinkwater C, Moffatt A., S. B. Ways to Wellness, The First Six Years Approach, Findings and Learning. Ways to Wellness; 2021.

[6] Polley M, Seers H, Toye O, Henkin T, Waterson H, Bertotti M, Chatterjee HJ. Building the economic evidence case for social prescribing. London: National Academy for Social Prescribing; 2023.

[7] Bertotti M, Hayes D, Berry V, Jarvis-Beesley P, Husk K. Social prescribing for children and young people. Lancet Child Adolesc Health. 2022;6:835–7. 36206788 10.1016/S2352-4642(22)00248-6

[8] Cartwright L, Burns L, Akinyemi O, Carder-Gilbert H, Tierney S, Elston J, et al. Who is and isn’t being referred to social prescribing? London: National Academy for Social Prescribing; 2022.

[9] Rice R. The missing link: social prescribing for children and young people. Essex: Barnardos; 2023.

[10] Muhl C, Mulligan K, Bayoumi I, Ashcroft R, Ross-White A, Godfrey C. Social prescribing for children and youth: A scoping review protocol. PLoS One. 2024;19:e0297535. 38457470 10.1371/journal.pone.0297535 PMC10923428

[11] Bertotti M, Frostick C, Sharpe D, Temirov O. A two-year evaluation of the Young People Social Prescribing (YPSP) pilot. Institute for Connected Communities, University of East London. https://repository.uel.ac.uk/item/88x15; 2020.

[12] Hayes D, Jarvis-Beesley P, Mitchell D, Polley M., K. H, Collaborative]. ObotNAP. The impact of social prescribing on children and young people’s mental health and wellbeing. London: National Academy for Social Prescribing.; 2023.

[13] Adjei NK, Schluter DK, Straatmann VS, Melis G, Fleming KM, McGovern R, et al. Impact of poverty and family adversity on adolescent health: a multi-trajectory analysis using the UK Millennium Cohort Study. Lancet Reg Health Eur. 2022;13:100279. 35199082 10.1016/j.lanepe.2021.100279 PMC8841277

[14] Basu AP. Social prescribing: can it help disabled children? Developmental Medicine & Child Neurology 2021; 63: 1132-. 10.1111/dmcn.14973 34490621

[15] Morris C, Janssens A, Tomlinson R, Williams J, Logan S. Towards a definition of neurodisability: a Delphi survey. Dev Med Child Neurol. 2013;55:1103–8. 23909744 10.1111/dmcn.12218

[16] Kuo DZ, Cohen E, Agrawal R, Berry JG, Casey PH. A national profile of caregiver challenges among more medically complex children with special health care needs. Arch Pediatr Adolesc Med. 2011;165:1020–6. 22065182 10.1001/archpediatrics.2011.172 PMC3923457

[17] Ostojic K, Karem I, Paget SP, Berg A, Dee-Price BJ, Lingam R, et al. Social determinants of health for children with cerebral palsy and their families. Dev Med Child Neurol. 2024;66:32–40. 37179527 10.1111/dmcn.15640

[18] Paget SP, McIntyre S, Goldsmith S, Ostojic K, Shrapnel J, Schneuer F, et al. Non-attendance at outpatient clinic appointments by children with cerebral palsy. Dev Med Child Neurol. 2022;64:1106–13. 35244200 10.1111/dmcn.15197 PMC9545710

[19] Pozniak K, King G, Chambers E, Martens R, Earl S, Kraus de Camargo O, et al. What do parents want from healthcare services? Reports of parents' experiences with pediatric service delivery for their children with disabilities. Disabil Rehabil 2023: 1–14. 10.1080/09638288.2023.2229733 37419932

[20] Lach LM, Kohen DE, Garner RE, Brehaut JC, Miller AR, Klassen AF, Rosenbaum PL. The health and psychosocial functioning of caregivers of children with neurodevelopmental disorders. Disabil Rehabil. 2009;31:607–18. 19360498 10.1080/09638280802242163

[21] Gordon K, Gordon L, Basu AP. Social prescribing for children and young people with neurodisability and their families initiated in a hospital setting: a systematic review. BMJ Open. 2023;13:e078097. 38135327 10.1136/bmjopen-2023-078097 PMC11148699

[22] Fritz CQ, Thomas J, Gambino J, Torok M, Brittan MS. Prevalence of social risks on inpatient screening and their impact on pediatric care use. Hosp Pediatr. 2020;10:859–66. 32967923 10.1542/hpeds.2020-0094

[23] Brennan L, Stres DP, Egboko F, Patel P, Broad E, Brewster L, et al. How do children’s hospitals address health inequalities: a grey literature scoping review. BMJ Open. 2024;14:e079744. 38171615 10.1136/bmjopen-2023-079744 PMC10773373

[24] Bryson I, Tredgett E, AP B. Hospital based social prescribing: Investigating parental views on the proposed Introduction of link workers for children with disabilities. Developmental Medicine & Child Neurology: Wiley; 2022. 68–9.

[25] Skivington K, Matthews L, Simpson SA, Craig P, Baird J, Blazeby JM, et al. A new framework for developing and evaluating complex interventions: update of medical research council guidance. BMJ. 2021;374:n2061. 34593508 10.1136/bmj.n2061 PMC8482308

[26] NHS. How to care for children with complex needs. https://www.nhs.uk/conditions/social-care-and-support-guide/caring-for-children-and-young-people/how-to-care-for-children-with-complex-needs/ (Accessed 24.12.24 2024)

[27] Berry JG, Hall M, Cohen E, O’Neill M, Feudtner C. Ways to identify children with medical complexity and the importance of why. J Pediatr. 2015;167:229–37. 26028285 10.1016/j.jpeds.2015.04.068 PMC5164919

[28] Hoffmann TC, Glasziou PP, Boutron I, Milne R, Perera R, Moher D, et al. Better reporting of interventions: template for intervention description and replication (TIDieR) checklist and guide. BMJ. 2014;348:g1687. 24609605 10.1136/bmj.g1687

[29] Good A. Outcomes Star™ Psychometric Factsheet: Support Star (Young People)™. Triangle 2020.

[30] Stevens K. Developing a descriptive system for a new preference-based measure of health-related quality of life for children. Qual Life Res. 2009;18:1105–13. 19693703 10.1007/s11136-009-9524-9

[31] Stevens K. Valuation of the Child Health Utility 9D Index. Pharmacoeconomics. 2012;30:729–47. 22788262 10.2165/11599120-000000000-00000

[32] Ratcliffe J, Flynn T, Terlich F, Stevens K, Brazier J, Sawyer M. Developing adolescent-specific health state values for economic evaluation: an application of profile case best-worst scaling to the Child Health Utility 9D. Pharmacoeconomics. 2012;30:713–27. 22788261 10.2165/11597900-000000000-00000

[33] Feng YS, Kohlmann T, Janssen MF, Buchholz I. Psychometric properties of the EQ-5D-5L: a systematic review of the literature. Qual Life Res. 2021;30:647–73. 33284428 10.1007/s11136-020-02688-y PMC7952346

[34] McNamara S, Schneider PP, Love-Koh J, Doran T, Gutacker N. Quality-adjusted life expectancy norms for the english population. Value Health. 2023;26:163–9. 35965226 10.1016/j.jval.2022.07.005

[35] Casey P, Patalay P, Deighton J, Miller SD, Wolpert M. The Child Outcome Rating Scale: validating a four-item measure of psychosocial functioning in community and clinic samples of children aged 10–15. Eur Child Adolesc Psychiatry. 2020;29:1089–102. 31659441 10.1007/s00787-019-01423-4

[36] Casey P, Patalay P, Deighton J, Miller SD, Wolpert M. The Child Outcome Rating Scale: validating a four-item measure of psychosocial functioning in community and clinic samples of children aged 10–15. Eur Child Adolesc Psychiatry. 2020;29:1089–102. 31659441 10.1007/s00787-019-01423-4

[37] Tennant R, Hiller L, Fishwick R, Platt S, Joseph S, Weich S, et al. The Warwick-Edinburgh Mental Well-being Scale (WEMWBS): development and UK validation. Health Qual Life Outcomes. 2007;5:63. 18042300 10.1186/1477-7525-5-63 PMC2222612

[38] U.S. Bureau of Labor Statistics. National Longitudinal Surveys. https://www.nlsinfo.org/content/cohorts/nlsy79-children/topical-guide/income/financial-strain. Accessed 24 Dec 2024.

[39] Braun V, Clarke V. Thematic Analysis: A Practical Guide. SAGE Publications; 2021.

[40] Nicholls J, Lawlor E, Neitzert E, Goodspeed T. Guide to Social Return on Investment. UK: SROI Network, Office of the Third Sector; 2012.

[41] Community Investment and Homelessness Values from the Social Value Bank. HACT and Simetrica; 2018.

[42] Trotter L, Vine J, Leach M, Fujiwara D. Measuring the social impact of community investment: a guide to using the Wellbeing Valuation approach,. Housing Associations’ Charitable Trust (HACT). 2014.

[43] Jones C, Hartfiel N, Brocklehurst P, Lynch M, Edwards RT. Social return on investment analysis of the health precinct community hub for chronic conditions. Int J Environ Res Public Health. 2020;17(14):5249. 32708127 10.3390/ijerph17145249 PMC7399792

[44] Skinner A, Hartfiel N, Lynch M, Jones AW, Edwards RT. Social return on investment of social prescribing via a diabetes technician for preventing type 2 diabetes progression. Int J Environ Res Public Health. 2023;20(12):6074. 37372661 10.3390/ijerph20126074 PMC10298404

[45] Makanjuola A, Lynch M, Hartfiel N, Cuthbert A, Wheeler HT, Edwards RT. A social return on investment evaluation of the pilot social prescribing EmotionMind dynamic coaching programme to improve mental wellbeing and self-confidence. Int J Environ Res Public Health. 2022;19(17):10658. 36078373 10.3390/ijerph191710658 PMC9518074

[46] Lloyd E. The Social Impact of the Arfon Social Prescription Model Social Return on Investment (SROI) Evaluation and Forecast Report. Cardiff: Social Value Cymru; 2018.

[47] Envoy Partnership. Self-Care social prescribing. London: Kensington & Chelsea Social Council and NHS West London Clinical Commissioning Group; 2018. https://www.kcsc.org.uk/sites/default/files/civicrm/persist/contribute/files/Self%20Care/7641_SROI-Report_DIGITAL_AW.pdf.

[48] Hutchinson CL, Berndt A, Forsythe D, Gilbert-Hunt S, George S, Ratcliffe J. Valuing the impact of health and social care programs using social return on investment analysis: how have academics advanced the methodology? A systematic review Bmj Open. 2019;9:e029789. 31446413 10.1136/bmjopen-2019-029789 PMC6720245

[49] Government U. Disability Living Allowance (DLA) for adults. https://www.gov.uk/dla-disability-living-allowance-benefit/DLA-rates (Accessed 24.12.24 2024)

[50] Leo's Neonatal. https://leosneonatal.org/ (accessed 24.12.24 2024)

[51] Government U. Carers' Allowance. https://www.gov.uk/carers-allowance (Accessed 24.12.24 2024)

[52] Trotter L, Rallings Adams M-K. Valuing improvements in mental health: Applying the wellbeing valuation method to WEMWBS. London, UK.: HACT.;2017.

[53] Ayers KB, Riddle I. Implications of health equity for children with disabilities. JAMA Pediatr. 2024;178:518–9. 38683627 10.1001/jamapediatrics.2024.0763

[54] Neshan M, Padmanaban V, Tsilimigras DI, Obeng-Gyasi S, Fareed N, Pawlik TM. Screening tools to address social determinants of health in the United States: A systematic review. J Clin Transl Sci. 2024;8:e60. 38655456 10.1017/cts.2024.506 PMC11036426

[55] Garg A, Toy S, Tripodis Y, Silverstein M, Freeman E. Addressing social determinants of health at well child care visits: a cluster RCT. Pediatrics. 2015;135:e296–304. 25560448 10.1542/peds.2014-2888 PMC4306802

[56] Messmer E, Brochier A, Joseph M, Tripodis Y, Garg A. Impact of an on-site versus remote patient navigator on pediatricians’ referrals and families’ receipt of resources for unmet social needs. J Prim Care Community Health. 2020;11:2150132720924252. 32449443 10.1177/2150132720924252 PMC7249580

[57] Gottlieb LM, Hessler D, Long D, Laves E, Burns AR, Amaya A, et al. Effects of Social needs screening and in-person service navigation on child health: a randomized clinical trial. JAMA Pediatr. 2016;170:e162521. 27599265 10.1001/jamapediatrics.2016.2521

[58] Patty NJS, van Meeteren KM, Willemen AM, Mol MAE, Verdonk M, Ketelaar M, Schuengel C. Understanding burnout among parents of children with complex care needs: a scoping review followed by a stakeholder consultation. J Child Fam Stud. 2024;33:1378–92.

[59] Thomson J, Shah SS, Simmons JM, Sauers-Ford HS, Brunswick S, Hall D, et al. Financial and social hardships in families of children with medical complexity. J Pediatr. 2016;172(187–93):e1. 10.1016/j.jpeds.2016.01.049 PMC4846519 26897040

[60] Bertotti M, Frostick C, Hutt P, Sohanpal R, Carnes D. A realist evaluation of social prescribing: an exploration into the context and mechanisms underpinning a pathway linking primary care with the voluntary sector. Prim Health Care Res Dev. 2018;19:232–45. 29215328 10.1017/S1463423617000706 PMC5904290

[61] Simpson S, Furlong M, Giebel C. Exploring the enablers and barriers to social prescribing for people living with long-term neurological conditions: a focus group investigation. BMC Health Serv Res. 2021;21:1230. 34774034 10.1186/s12913-021-07213-6 PMC8590354

[62] Moore C, Unwin P, Evans N, Howie F. Social prescribing: Exploring general practitioners’ and healthcare professionals’ perceptions of, and engagement with, the NHS model. Health Soc Care Community. 2022;30:e5176–85. 35869824 10.1111/hsc.13935 PMC10084047

[63] Ayorinde A, Grove A, Ghosh I, Harlock J, Meehan E, Tyldesley-Marshall N, et al. What is the best way to evaluate social prescribing? A qualitative feasibility assessment for a national impact evaluation study in England. J Health Serv Res Policy. 2024;29:111–21. 38101334 10.1177/13558196231212854 PMC10910745

[64] Fixsen A, Seers H, Polley M, Robins J. Applying critical systems thinking to social prescribing: a relational model of stakeholder “buy-in.” BMC Health Serv Res. 2020;20:580. 32580718 10.1186/s12913-020-05443-8 PMC7312116

[65] Ways to Wellness. SPACE PILOT videos. 2024. https://www.youtube.com/@ways2wellnessUK.

[66] Brewster L, Brennan L, Hindocha A, Lunn J, Isba R. Understanding responsibility for health inequalities in children’s hospitals in England: a qualitative study with hospital staff. BMJ Open. 2024;14:e081056. 38604623 10.1136/bmjopen-2023-081056 PMC11015292

[67] Hayes D, Olsson A, Begum S, Bertotti M, Jarvis-Beesley P, Stapley E. Barriers and facilitators to social prescribing in child and youth mental health: perspectives from the frontline. Eur Child Adolesc Psychiatry. 2024;33:1465–79. 37405485 10.1007/s00787-023-02257-x PMC11098893

