# Supplementary figures and images for: Hospital in-reach family-centred social prescribing pilot for children with neurodisability: mixed methods evaluation with social return on investment analysis
Source: BMC Health Serv Res. 2025 Jan 30;25:176. doi: 10.1186/s12913-025-12329-0 (PMC11781045; doi:10.1186/s12913-025-12329-0)

# Figure S1

Social Return On Investment Logic Model

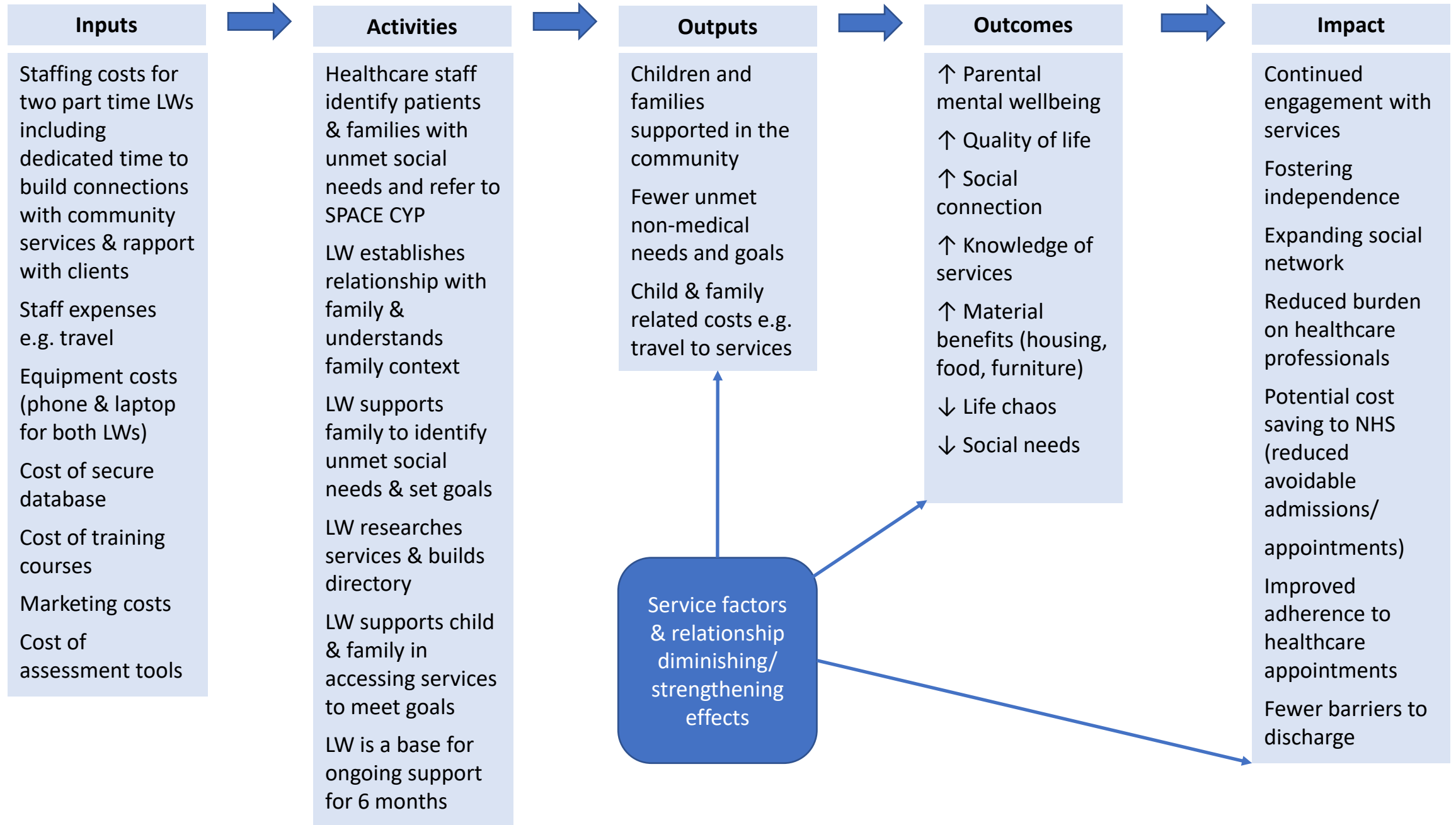

Supplement: Supplementary file 1 — Supplementary Material 1: Figure S1: SROI logic model. [file 12913_2025_12329_MOESM1_ESM.pdf]
