# Supplementary material for: Hospital in-reach family-centred social prescribing pilot for children with neurodisability: mixed methods evaluation with social return on investment analysis
Source: BMC Health Serv Res. 2025 Jan 30;25:176. doi: 10.1186/s12913-025-12329-0 (PMC11781045; doi:10.1186/s12913-025-12329-0)
Supplement: Supplementary file 2 — Supplementary Material 2. S2: Topic guides for qualitative interviews. [file 12913_2025_12329_MOESM2_ESM.pdf]

Supplementary data S2: Topic guides for qualitative interviews.

A) Parents. B) Link workers. C) Healthcare professionals.

A) Interview topic guide, SPACE CYP (parents)

Note: The interview schedule is developmental. The questions will need to be specifically tailored to the individual interviewee.

<u>Introduction</u>

- Thank participant for attending.
- Explain the purpose of this interview: to understand their experience of the study and gain feedback.
    - No right or wrong answers.
- Explain interview will be audio recorded but details will be kept confidential, and recording will be anonymised.
- OK to use photos collected as points of discussion as previously proposed?
- Any questions or concerns?

Ideally interviews done with the child present and use the photos to help the child feed back and engage. Structure for using the photos as prompts is "SHOWED".

- 1. What do you **S**ee here?
- 2. What is really **H**appening here?
- 3. How does this relate to **O**ur lives?
- 4. **W**hy does this condition **E**xist?
- 5. What can we **D**o about it?

<u>First interview</u>

- To understand something about the child, things they like to do, things they struggle with
- Experiences of meeting with link worker
- Services referred to.
- Did they access the services? If not, why not
- Was LW support given/needed to access services?
- Experience of those services (If any observations done, can talk about relevant findings and sense check interpretation)
- Has anything changed as a result?
- Experience of ongoing contact with link worker
- Is there anything they would change about the way the service is run?

Second interview

- Researcher will need to refer back to the first interview prior to undertaking the second interview and pick up on any relevant points to pursue.
- Again, use photos as basis to prompt discussion around meaningful moments e.g. child engaging in services referred to.
- Have any needs been addressed or partially addressed? What needs remain? Was 6 months a useful period of time for LW involvement?
- What services is the child accessing now that they weren’t before? Will they continue after the end of the project? If not, why not?
- Experience of those services (If any observations done, can talk about relevant findings and sense check interpretation)
- Was there any tension between needs of the child vs needs of other family members?
- Do the family feel they have learned more about how to access services (getting at transferable skills, gradual reduction in need for external support etc.) as a result of LW support? If help is still needed, do they feel they know where to get it?
- What were the best things about being involved in the project?
- What were the worst things about being involved in the project?
- Is there anything they think it would be better to do differently? If so, find out what and how.

Feedback

- Thank you.
- Are we asking the right questions?
- Is there anything else you think will be useful for us to know?

B) Interview topic guide, SPACE CYP (link workers)

Note: The interview schedule is developmental. The questions will need to be specifically tailored to the individual interviewee.

Introduction

- Thank participant for attending.
- Explain the purpose of this interview: to understand their experience of the study and gain feedback.
    - No right or wrong answers.
- Explain interview will be audio recorded but details will be kept confidential, and recording will be anonymised.
- Any questions or concerns?

Interview main body.

- Experiences of meeting with children and families
- Services referred to, and any challenges.
- Were the right type of services available to meet needs?
- Did the families access the services? If not, why not
- Was LW support given/needed to access services?
- Experience of ongoing contact with children and families
- Overall perception of benefit from the LW service
- Biggest challenges
- Any additional support/training that would be helpful.
- Is there anything they would change about the way the service is run?
- Was there any tension between needs of the child vs needs of other family members?

Feedback

- Thank you.
- Are we asking the right questions?
- Is there anything else you think will be useful for us to know?

C) Interview topic guide SPACE CYP (healthcare professionals)

Note: The interview schedule is developmental. The questions will need to be specifically tailored to the individual interviewee.

Introduction

- Thank participant for attending.
- Explain the purpose of this interview: to understand their perspective of the study, and gain feedback and insight to broaden scope.
    - No right or wrong answers.
- Explain interview will be audio recorded but details will be kept confidential, and recording will be anonymised.
- Any questions or concerns?

Interview main body.

- Area of work of the HCP involved – what job do they do.
- Have they come across SP in the past? In what context?
    - (if not, explain SP in broad terms)
- Are they aware of the GNCH SP pilot for children with neurodisability?
  (if so, in what context, and any perceptions/experience)
  (if not, explain SP pilot)
- Are they aware of other similar services in the hospital e.g. in other children's specialties?
- Do they think a similar type of service would be valuable for the children they are involved in looking after? What would that service look like? What kinds of non-medical needs would need to be addressed? what challenges would they foresee in delivering a service like this?
- Any other thoughts from them?

Feedback

- Thank you.
- Are we asking the right questions?
- Is there anything else you think will be useful for us to know?
